# Quality of life and patient satisfaction in bracing treatment of adolescent idiopathic scoliosis

**DOI:** 10.1186/s13013-018-0172-0

**Published:** 2018-12-14

**Authors:** Lucas Piantoni, Carlos A. Tello, Rodrigo G. Remondino, Ernesto S. Bersusky, Celica Menéndez, Corina Ponce, Susana Quintana, Felisa Hekier, Ida A. Francheri Wilson, Eduardo Galaretto, Mariano A. Noël

**Affiliations:** 1 Servicio de Patología Espinal, Hospital de Pediatría Prof. Dr. Juan P. Garrahan, Combate de los Pozos 1881. C1245AAM, CABA, Buenos Aires, Argentina; 2 Departamento de Salud Mental, Hospital de Pediatría Prof. Dr. Juan P. Garrahan, Buenos Aires, Argentina; 3 Departamento de Servicio Social, Hospital de Pediatría Prof. Dr. Juan P. Garrahan, Buenos Aires, Argentina

**Keywords:** Adolescent idiopathic scoliosis, Orthosis, Brace, HRQoL, Satisfaction, Non-surgical treatment of scoliosis

## Abstract

**Background:**

Bracing is used as a valid non-surgical treatment for adolescent idiopathic scoliosis (AIS) to avoid progression of the deformity and thereby surgery. The effect of bracing treatment on quality of life of patients with AIS has been a topic of interest in the international literature. The aim of this study was to evaluate the quality of life and patient satisfaction during bracing treatment for AIS of a pediatric hospital.

**Material and method:**

We assessed a total of 43 non-consecutive female patients (mean age at questionnaire, 13 years and 1 month and 10 years and 8 months to 14 years and 5 months; mean period of usage of brace, 1 year and 7 months), with adolescent idiopathic scoliosis (AIS), older than 10 years of age until skeletal maturity, with a Risser sign less than 3 and scoliosis between 20 and 45°, treated with thoracolumbosacral orthosis (TLSO) for a period longer than 6 months, and without other comorbidities or previous surgeries, were evaluated. The patients were administered a previously validated to Spanish questionnaire on quality of life (Brace Questionnaire (BrQ); Grivas TB et al.). BrQ is a validated tool and is considered a disease-specific instrument; its score ranges from 20 to 100 points, and higher BrQ scores are associated with better quality of life.

**Results:**

The patients reported using the brace for a mean of 17.6 h daily and for a mean period of 1 year and 7 months at the time of the study. Overall, 72% of the study population reported to be in some way psychologically affected by the brace wearing, 56% felt their basic motor activities were affected, 54% felt socialization with their environment was affected, 46% considered their quality of life deteriorated due to pain, and 40% reported conflicts in the school environment.

**Conclusion:**

Patients with AIS treated with bracing reported a negative impact (53.5% overall) on quality of life and treatment satisfaction in terms of psychological, motor, social, and school environment aspects. An interdisciplinary approach would be important for the integrated psychosocial care of these patients.

## Introduction

Bracing is used as a valid conservative treatment for adolescent idiopathic scoliosis (AIS) to avoid progression of the deformity and thereby surgery [1–9, 14–16, 19–24, 26–29, 33, 40, 41, 43, 50, 51, 58]. In 1946, Blount and Schmidt developed a brace to be used postoperatively, subsequently known as the Milwaukee brace [80]. Years later, the Boston brace was developed by Hall and Miller. Other braces are as follows: the Charleston, the Providence, the thoracolumbosacral orthosis (TLSO), the Cheneau-Rigo, Lyonnais, and others [81].

The etiology of AIS has a strong genetic component [13, 18, 33], with different patterns of inheritance [37, 47]. Additionally, multiple non-genetic causes have been associated with the development of AIS [13].

The classic TLSO brace is indicated in patients with AIS aged between 10 and 15 years, approximately, with a Risser sign less than 3, that is pre-menarche or with onset of menarche less than 1 year, have curves between 25 and 45° or of 20° with a progression greater than 5° since the last control, and an apex at T7 or below [1–3].

The quality of life and treatment satisfaction of the patient with AIS undergoing bracing is a subject of interest in the international literature [1, 10–15, 17–19, 25, 48, 49, 55, 57, 59–63, 76–78, 86]. The natural course of AIS has been found to be extremely variable [12, 13, 18, 33, 60–62], and the effectivity rate of bracing treatment has historically been difficult to quantify. Nevertheless, bracing has been shown to be an effective non-surgical treatment method in different studies [1, 44–46].

Recent studies have focused on the assessment of the quality of life of the patients related to hours of daily use of the brace and long-term results [27–31, 36]. Other studies have evaluated the use of the brace and the outcome of the treatment associated with health, self-esteem, and quality of life [1, 64–75].

Our hypothesis is that nonetheless, the orthosis treatment must be indicated whenever possible according to the inclusion criteria, this paper highlights the importance of health-related quality of life (HRQoL) measurement in assessing how AIS patients perceive the impact of their deformity condition and the need of multi-disciplinary observation and treatment.

There is no report in published literature about bullying situation in AIS children with an orthosis, and this was one of the trigger points that starts this AIS and orthosis paper. The aim of this study was to assess the quality of life and treatment satisfaction of the patient undergoing bracing treatment for AIS of a pediatric hospital.

## Material and method

Forty-three non-consecutive female patients undergoing bracing treatment for AIS were assessed. The inclusion criteria were female sex, patients between 10 years of age to skeletal maturity, Risser sign less than or equal to 3, scoliosis between 20 and 45°, undergoing bracing treatment with the TSLO without electronic monitoring control device for a period of at least 6 months, and without comorbidities or previous treatment or thoracolumbar surgery. The patients were administered a questionnaire on quality of life related to AIS and bracing treatment (Brace Questionnaire (BrQ); Grivas et al.) consisting of 34 questions with 5 options each [56], delivered by a staff member at the end of the appointment who stayed present during the completion of the questionnaire (15 min approximately). Every patient’s visit included a clinical exam and X-ray exam; they took place every 6 months and were assessed by two different spine surgeons. Mean age at questionnaire submission was 13 years and 1 month (range, 10 years and 8 months to 14 years and 5 months). Mean period of the term of usage of brace is 1 year and 7 months (7 months to 3 years and 4 months) at questionnaire submission.

We used the English version of the paper “Development and preliminary validation of Brace Questionnaire (BrQ): a new instrument for measuring the quality of life of brace-treated scoliotics” [56]. Translation to Spanish, subsequent translation to the English language, and subsequent translation to Spanish was conducted by three different specialists, and validity and reliability procedures were conducted. Thereafter, the Director of Education and Research Department at the hospital delivered the validated questionnaire in Spanish in order to be used with our pediatric population.

The following demographic aspects were recorded: age at onset of bracing treatment, months or years of brace wearing, and mean hours of daily brace wearing. Additionally, the following issues were assessed: if the brace made the patient feel ill or made the patient feel tired when walking; if the patient was able to put on and take off the brace by themselves; if the brace interfered with eating, sleeping, or breathing; if the brace made the patient feel nervous or worried; if the patient believed his/her life would be better without the brace; if he/she felt the treatment was beneficial; if the brace wearing caused difficulties at school; if the brace caused pain and analgesics would be necessary; if he/she felt different compared from peers; if the brace caused problems in the family; and if his/her relationship with family and friends would be better without the brace wearing. For each of the questions, only one answer was given. To facilitate a data analysis, the survey was subdivided into five different domains referring to psychological, motor, school, pain, and socializing aspects.

BrQ is a validated, a disease specific instrument; it works with a score that ranges from 20 to 100 points, and higher BrQ scores would be associated with better quality of life (Table 1). This study was previously approved by the IRB of the hospital (Hospital de Pediatría Prof. Dr. Juan P. Garrahan Ethical Committee), approval serial # 870/2015.

**Table 1:** Brace Questionnaire (BrQ) administered to the patient

This questionnaire asks how you feel about your health, while you are wearing a brace. This is not a test and there are no right or wrong answers.	
Please read carefully every question	
Choose the best answer and mark with an x	
Example	
• During the last week, you were in a good mood for studying	
• Never	
• Almost never	
• Sometimes	
• Most of the times	
• Always	
Please tell us a few things about yourself:	
You are a boy/a girl (cross out what is NOT correct)	
How old are you? ......... years.	
You are wearing the brace since ………. months/years.	
You are wearing the brace for ….. hours/day	
Date ……………………………	
During the first 3 months	
1. The brace made you feel ill	
• Never	
• Almost never	
• Sometimes	
• Most of the time	
• Always	
2. You were afraid that your back will get worse	
• Never	
• Almost never	
• Sometimes	
• Most of the time	
• Always	
During the past 3 months while you were wearing the brace...	
3. You felt tired when walking	
• Never	
• Almost never	
• Sometimes	
• Most of the time	
• Always	
4. You were able to run	
• Never	
• Almost never	
• Sometimes	
• Most of the time	
• Always	
5. You managed to wear the brace without any help	
• Never	
• Almost never	
• Sometimes	
• Most of the time	
• Always	
6. You managed to take off the brace without any help	
• Never	
• Almost never	
• Sometimes	
• Most of the time	
• Always	
7. You could not eat well	
• Never	
• Almost never	
• Sometimes	
• Most of the time	
• Always	
8. You could not sleep well	
• Never	
• Almost never	
• Sometimes	
• Most of the time	
• Always	
9. You could not breathe well	
• Never	
• Almost never	
• Sometimes	
• Most of the time	
• Always	
During the past 3 months…	
10. The brace made you feel nervous	
• Never	
• Almost never	
• Sometimes	
• Most of the time	
• Always	
11. You felt worried because of the brace	
• Never	
• Almost never	
• Sometimes	
• Most of the time	
• Always	
12. You felt happy	
• Never	
• Almost never	
• Sometimes	
• Most of the time	
• Always	
13. You believed that your life would be better if you were not on brace	
• Never	
• Almost never	
• Sometimes	
• Most of the time	
• Always	
14. You believed that brace treatment was beneficial	
• Never	
• Almost never	
• Sometimes	
• Most of the time	
• Always	
During the past 1 month...	
15. You felt proud of yourself	
• Never	
• Almost never	
• Sometimes	
• Most of the time	
• Always	
16. You were satisfied with your body	
• Never	
• Almost never	
• Sometimes	
• Most of the time	
• Always	
During the past 1 month	
17. You felt strong and full of energy	
• Never	
• Almost never	
• Sometimes	
• Most of the time	
• Always	
18. You felt tired and exhausted because of the brace	
• Never	
• Almost never	
• Sometimes	
• Most of the time	
• Always	
During the past 1 month, because of the brace...	
19. You had difficulties with your lessons	
• Never	
• Almost never	
• Sometimes	
• Most of the time	
• Always	
20. You were absent from school	
• Never	
• Almost never	
• Sometimes	
• Most of the time	
• Always	
21. You found it hard to pay attention in the classroom	
• Never	
• Almost never	
• Sometimes	
• Most of the time	
• Always	
During the past 1 month, while you were wearing the brace...	
22. You had to take medication for pain	
• Never	
• Almost never	
• Sometimes	
• Most of the time	
• Always	
23. You had pain during the night	
• Never	
• Almost never	
• Sometimes	
• Most of the time	
• Always	
24. You had pain when walking	
• Never	
• Almost never	
• Sometimes	
• Most of the time	
• Always	
25. You had pain when sitting	
• Never	
• Almost never	
• Sometimes	
• Most of the time	
• Always	
26. You had pain when climbing stairs	
• Never	
• Almost never	
• Sometimes	
• Most of the time	
• Always	
27. You felt pins and needles in your arms or legs	
• Never	
• Almost never	
• Sometimes	
• Most of the time	
• Always	
During the past 1 month, because of the brace...	
28. You could not go out with your friends	
• Never	
• Almost never	
• Sometimes	
• Most of the time	
• Always	
29. Your friends felt compassion for you	
• Never	
• Almost never	
• Sometimes	
• Most of the time	
• Always	
30. You felt different from your peers	
• Never	
• Almost never	
• Sometimes	
• Most of the time	
• Always	
31. You had problems with your family	
• Never	
• Almost never	
• Sometimes	
• Most of the time	
• Always	
32. You believed that your relationship with your family or your friends would be better if you were not on brace	
• Never	
• Almost never	
• Sometimes	
• Most of the time	
• Always	
33. You stayed at home because you were ashamed	
• Never	
• Almost never	
• Sometimes	
• Most of the time	
• Always	
34. You wore special clothes	
• Never	
• Almost never	
• Sometimes	
• Most of the time	
• Always	

Reference [86]

## Results

In this study, we evaluated 43 non-consecutive female patients with AIS. They reported to have worn the TSLO brace for a mean of 17.6 h daily for a mean period of 1 year and 7 months (7 months to 3 years and 4 months), mean age at questionnaire of 13 years and 1 month (10 years and 8 months to 14 years and 5 months).

Based on the Brace Questionnaire, the following data was obtained:

- In the domain of psychology

Ninety percent of the patients stated that their life would be better without the brace, 86% did not feel happy, 82% did not feel proud of themselves during treatment, 81% felt tired when wearing the brace, 76% was afraid their curves would become worse, 73% stated to have difficulty being happy with their body image, 70% felt worried, 71% did not feel bracing treatment was entirely beneficial, 70% had difficulty feeling strong or full of energy, 63% felt nervous due to the brace wearing, 59% could not sleep well when wearing the brace, and 42% of the patients stated they felt ill during the bracing treatment.

- In the motor domain

Seventy-four percent of the patients felt tired when walking. Eating problems during brace wearing were reported by 51% of the patients. Overall, 50% stated to have some difficulties putting on and taking off the brace by themselves. Additionally, 49% of the patients had difficulty breathing during brace wearing.

- In the domain of social functioning

Seventy-five percent of the patients wore different clothes due to the brace, 69% felt their friends felt sorry for them, 57% felt different from their peers, 50% felt the relationship with their family and friends would be better if they were not wearing the brace, 42% had problems with their family due to the brace wearing, 42% sometimes stayed home because they felt ashamed of themselves, and 41% had difficulties socializing with their peers because of the brace.

- In the domain of pain

Fifty-eight percent had back pain when sitting, 55% had nightly back pain, 47% had pain when walking, 43% had back pain when climbing stairs, and 30% reported paresthesias in the upper and lower limbs.

- In the domain of school environment

Forty-eight percent of the study population at some moment had difficulties at school, 36% reported attention problems, and 35% were absent from school due to the brace wearing.

The analysis of the BrQ domain/question #4, 5, 6, 12, 14, 15, 16, and 17 reported a total main score of 63.2 over 100 points. Domain/question #1, 2, 3, 7, 8, 9, 10, 11, 13, 18, 19, 20, 21, 22, 23, 24, 25, 26, 27, 28, 29, 30, 31, 32, 33, and 34 resulted in a total main score of 65.4 over 100 points. Taking into account both subgroups, the BrQ standardized main score was 63.7 (41–94) over 100 points. A higher or better score would be associated with a better quality of life.

Even though no children reported bullying as an isolated or particular situation and the questionnaire does not include it as an explicit item, patients reported 57% felt different from their peers, 48% had difficulties at school, and 41% had difficulties socializing; therefore, we might strongly believe that bullying is an important issue needed to be addressed.

**Fig. 1:**
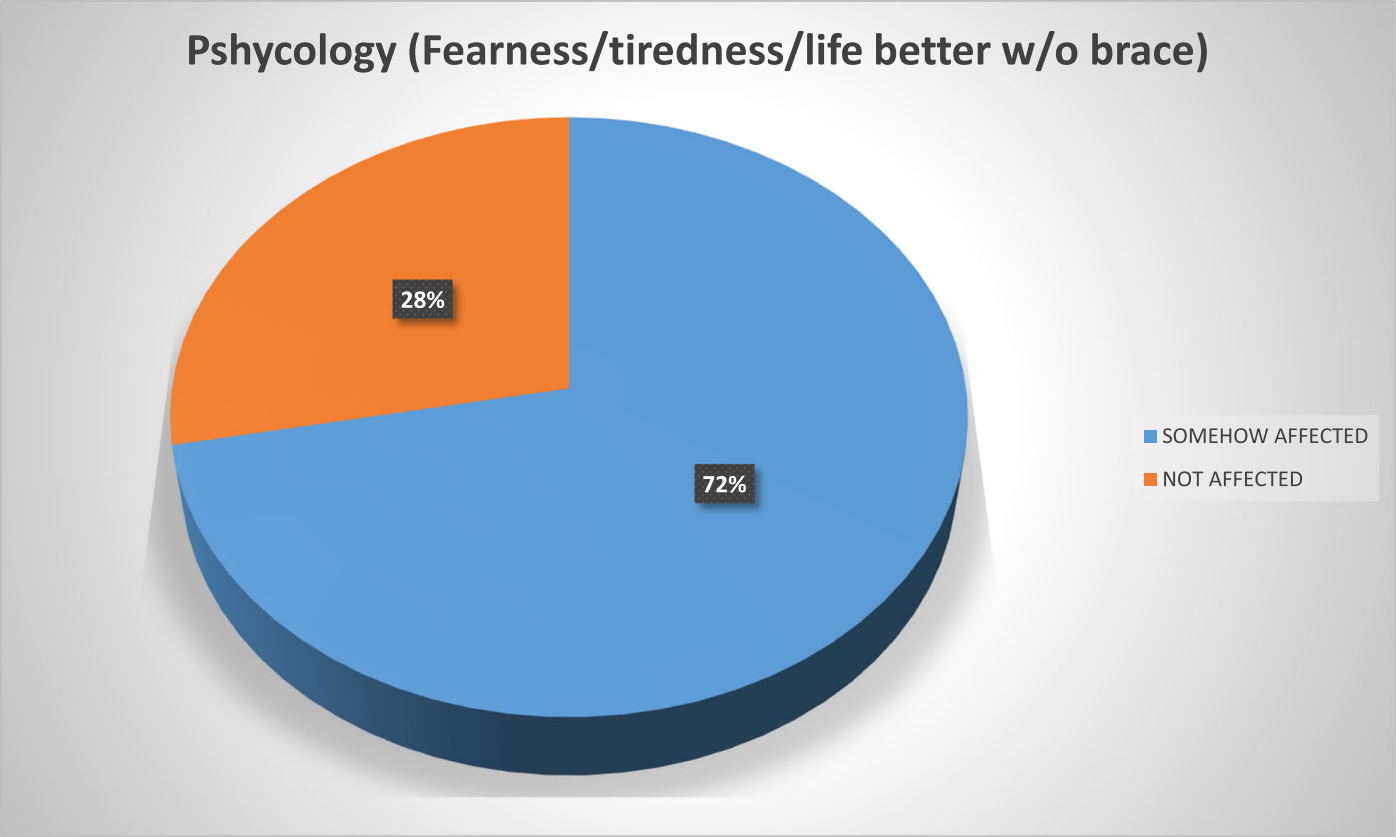
Psychology domain

**Fig. 2:**
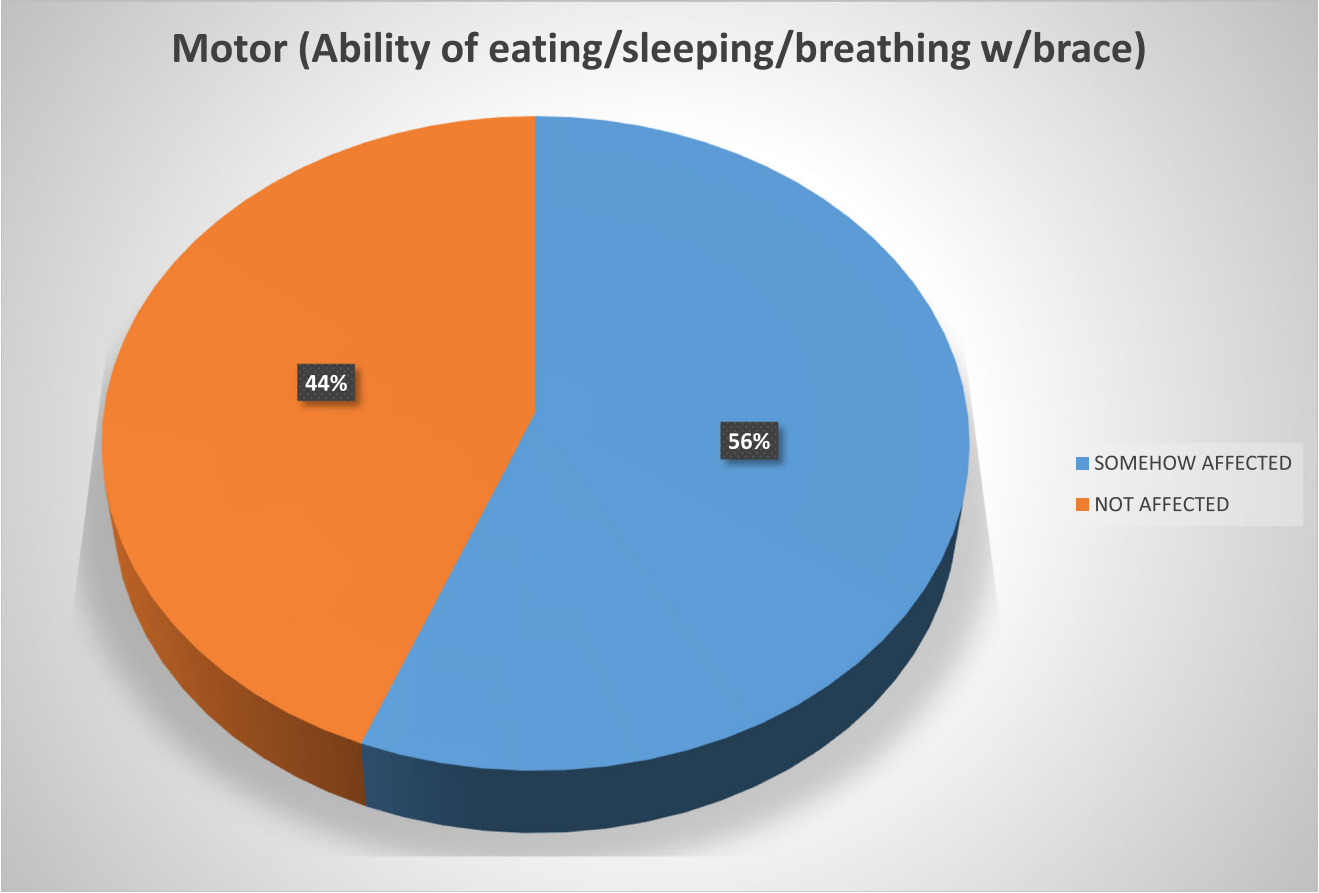
Motor domain

**Fig. 3:**
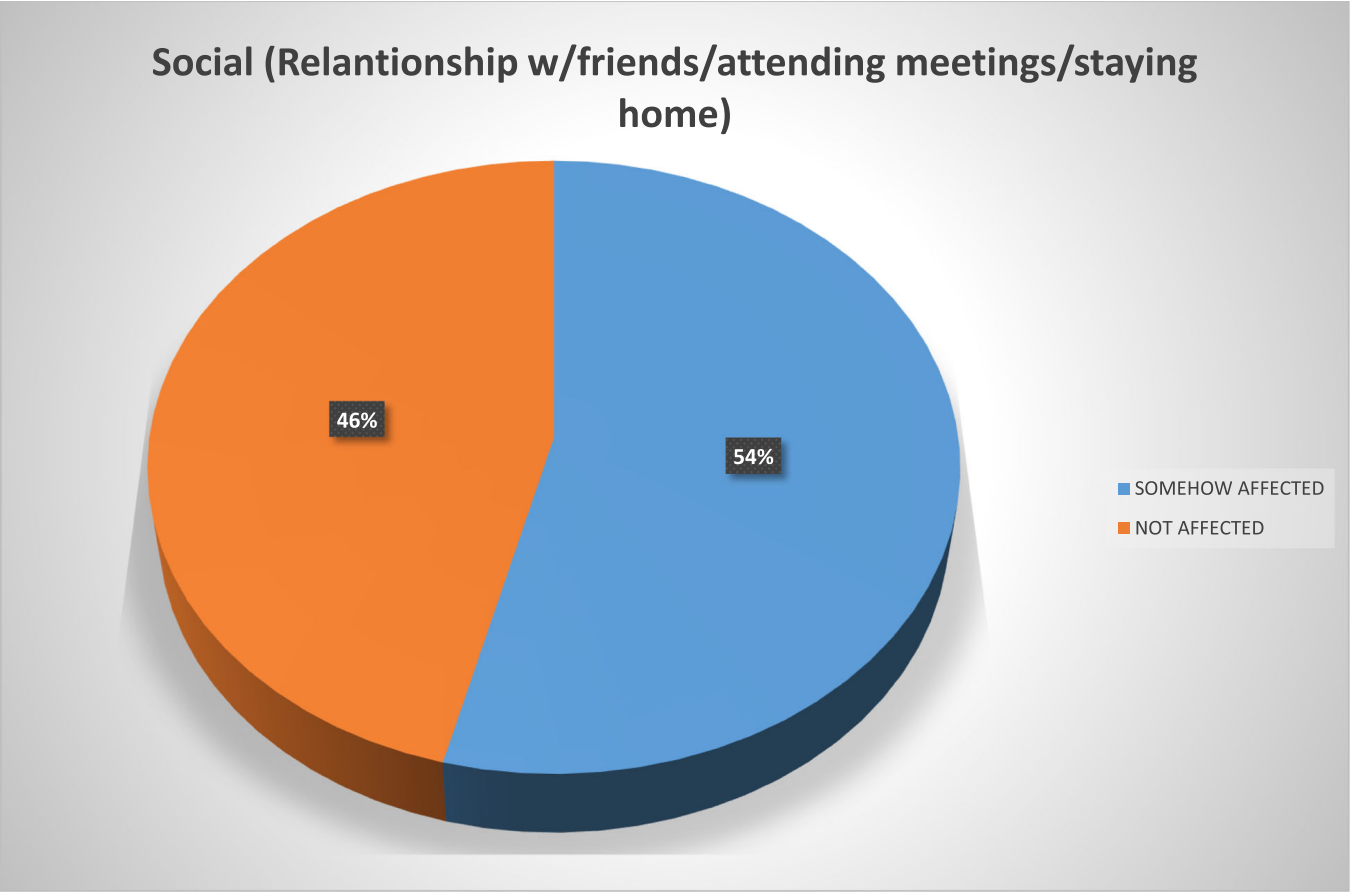
Social domain

**Fig. 4:**
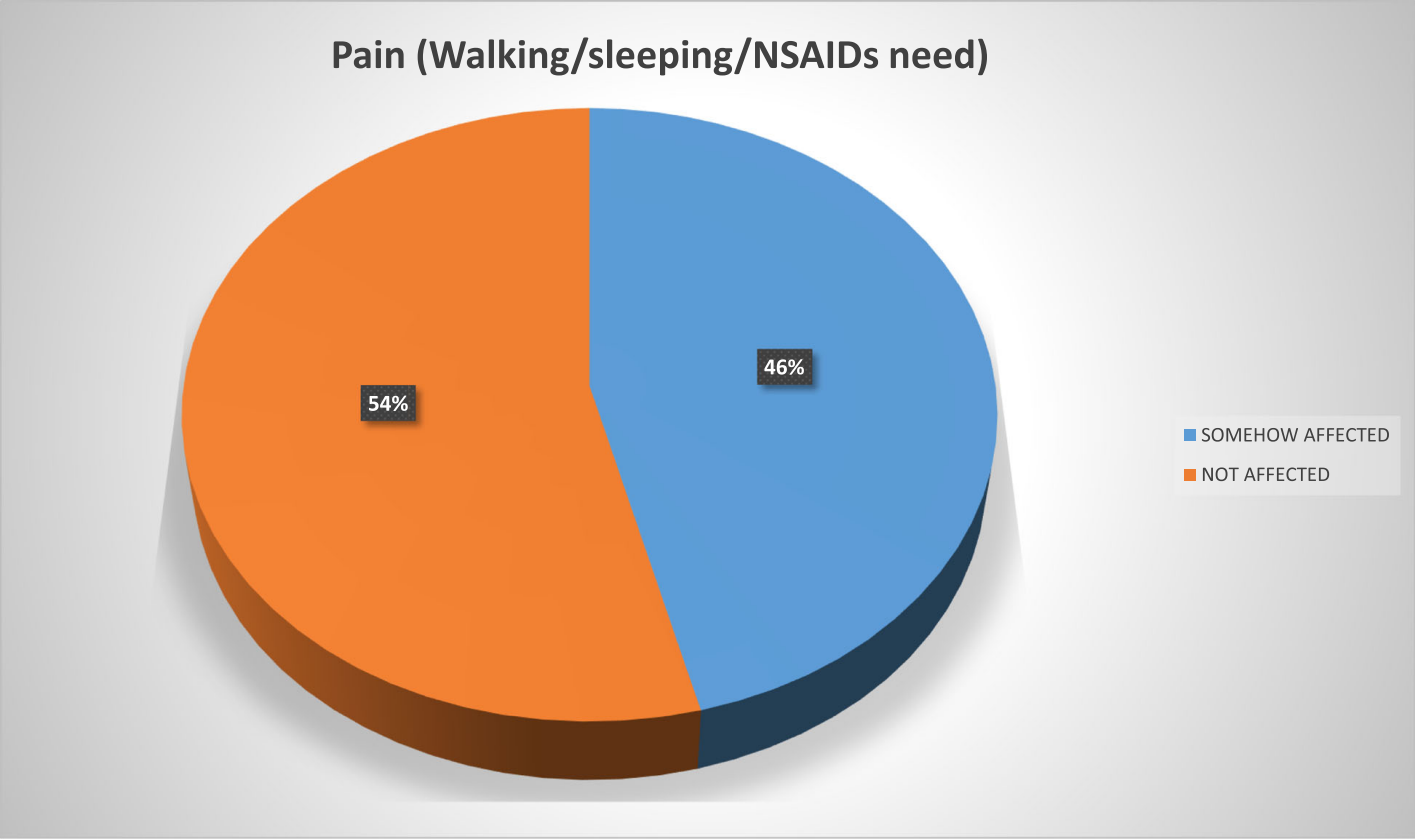
Pain domain

**Fig. 5:**
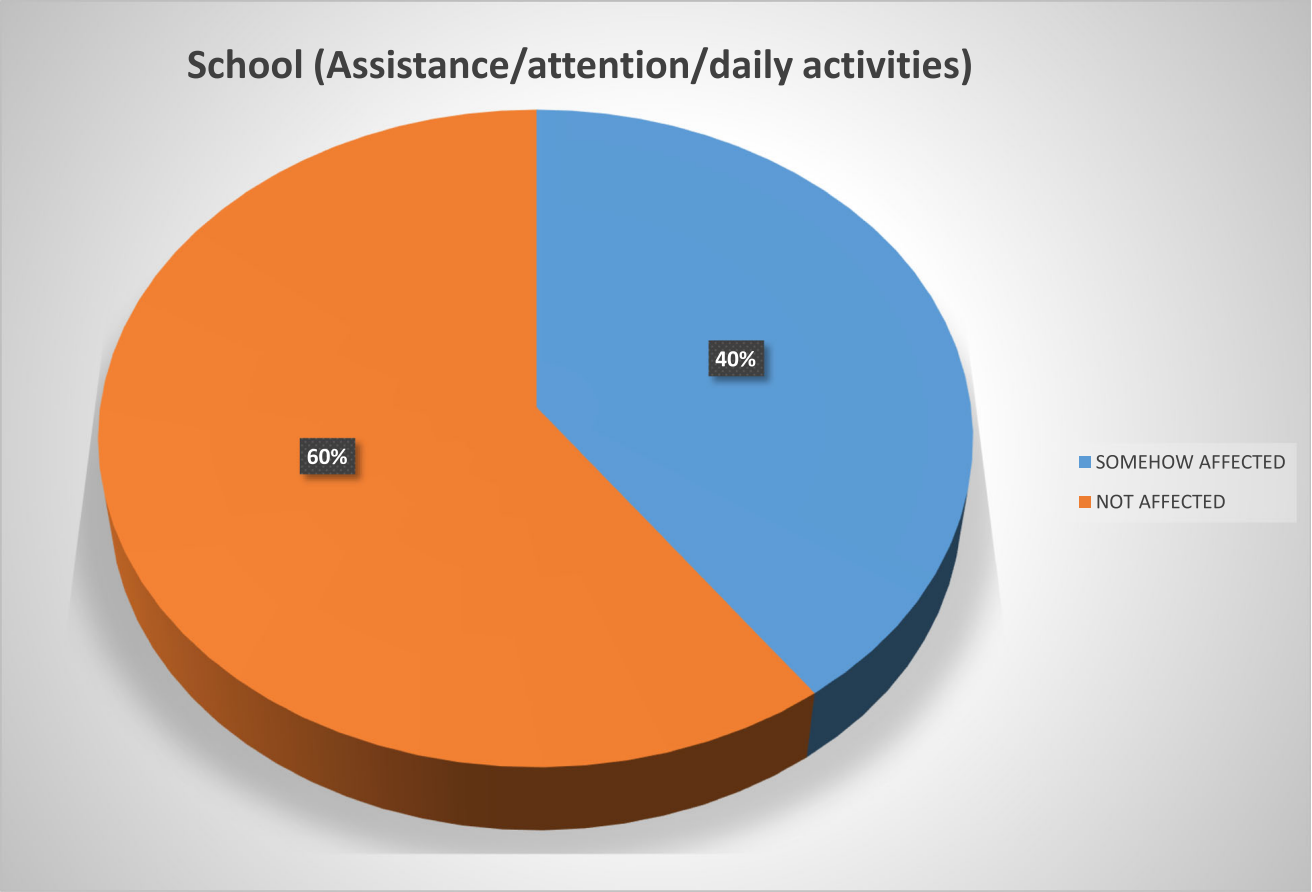
School domain

To ease the interpretation of the Brace Questionnaire answers, we developed two groups and tag them into “somehow affected” (“always”/“most of the time”/“sometimes”/“almost never” answers) and those considered “not affected” (“never” answer) (Figs. 1, 2, 3, 4, and 5).

## Discussion

Bracing it is known to be currently the standard of care for preventing curve progression and treating AIS. However, the effectiveness of bracing remains not totally clear, and it is unknown which adolescents in particular may benefit from bracing and which will be submitted to surgery [1]. We strongly support the use of brace when the inclusion criteria are present in AIS patients.

Brace wearing significantly affects the quality of life of the patients with AIS. Our study showed that patients with AIS treated with bracing reported a moderate negative impact on quality of life and treatment satisfaction in terms of psychological, motor, social, pain, and school environment aspects. Danielsson et al. and Kinel et al. showed that the use of the brace is associated with a social burden that may negatively affect the quality of life of the patient. Although they did not find any long-term changes in the health-related quality of life (HRQoL) questionnaire [17, 25, 62, 78, 79, 82], these authors as well as others confirm that patients who wear a brace are more concerned about their body image than those who undergo surgical treatment [11, 17, 38, 39, 42, 52, 66]. The same authors showed that the range of lumbar motion diminishes both in the brace-wearing group and in the group that underwent surgery (37% and 61%, respectively) [30, 32, 34, 35, 53, 54]. In our study, 54% of the entire population assessed felt that motor basic activities were somehow affected. On the other hand, Climent and Friedel et al. observed a considerable reduction in the HRQoL scores [76]. In our study, we assessed an important change in the domain of psychology, in which 90% of the patients stated that their lives would be better without wearing the brace, 82% did not feel proud of themselves using the brace, 81% felt tired when wearing the orthosis, 73% stated to have difficulty being happy with their body image, 70% felt worried, 63% reported to be nervous, and at least 42% reported to feel ill.

Lindeman et al. concluded that girls that were non-compliant to treatment had low self-esteem and did not seek social support from their peers or health professionals; had a poor body image, a low expectation of social success, and worse interaction with their environment; and experienced sleeping problems [83]. In our study, 69% felt their friends felt sorry for them, 57% felt different from their peers, 50% felt the relationship with their friends would be better if they were not using the brace, 42% sometimes stayed home because they felt ashamed of themselves, and 41% had difficulties socializing with their peers because of the brace.

In a study by Mc Lean et al., the psychological, functional, and family impact of brace treatment was evaluated in 31 patients with AIS. The initial brace-wearing period was described as stressful by 84%. Contact with peers in the same situation and the possibility of counseling on their condition were the variables mentioned by the families as the most important to help them to cope with the treatment-related stress [84]. Considering these findings and similar results in our population, our adolescent patients are offered support from the Department of Mental Health and Social Work.

Korovessis et al. compared patients with AIS undergoing brace treatment with controls. They reported loss of body flexibility, loss of friends, feeling ashamed of their body, being concerned about the future effect of the deformity on their body, and significantly more back pain [85]. Of the patients in our study, 72% reported problems during physical exercise, 50% had difficulty putting on or taking off the brace by themselves, 49% had breathing difficulties, and 40% felt ashamed of their body image.

In an update for the Scoliosis Research Society (SRS), Richards and Katz stated that the TLSO brace treatment continues being a challenge in adolescents, as psychological issues tend to make adolescents less compliant, while compliance is the most important factor for satisfactory results [79]. Our study showed that 72% of the adolescents felt psychologically affected by the brace wearing. Ninety percent of the patients stated that their life would be better without the brace, 82% did not feel proud of themselves during treatment, 73% stated to have difficulty being happy with their body image, 71% did not feel bracing treatment was entirely beneficial, 63% felt nervous due to the brace wearing, and 42% of the patients stated they felt ill during the bracing treatment.

Vasilaidis et al. demonstrated that health-related quality of life (HRQoL) refers to the patient’s ability to enjoy normal life activities, and that HRQoL variables are sometimes more important than the X-ray results or even pulmonary function tests. His study aimed to examine the impact of conservative treatment in HRQoL of the AIS patients. And we totally agree with his statements. Nonetheless, his scores in the domains of vitality and school activity were not affected, but in our study, we did see how they are affected somehow in a moderate to intense way. They concluded how vital is to highlight the importance of HRQoL measurement in assessing how AIS patients perceive the impact of their disease.

On the other hand, Simony et al. found better results regarding treatment satisfaction in surgically treated patients [49] and concluded that patients should be warned about the potential social and psychological impact of the treatment and that guidance is important in case the patient has to choose between surgical and non-surgical treatment [42, 66].

Finally, Grivas et al. concluded that the way to increase the knowledge on non-surgical treatment of AIS is to systematically analyze what is being done today to allow for comparisons in order to better understand the condition and how brace wearing psychologically affects our patients [40].

One of the limitations of our study was the small sample size compared to other studies. Additionally, there was a clear trend of parents trying to influence the answers of their child and even, albeit unsuccessfully, to answer for or correct the answers of their child. Therefore, a personalized interview with the child/adolescent would be important in addition to the interview with the parents.

## Conclusion

Patients with AIS treated with bracing reported a negative impact in our present study of 53.5%. Overall, 72% of our study population reported to be in some way psychologically affected by the brace wearing, 56% felt their basic motor activities were affected, 54% strongly considered their social functioning was affected, 46% felt their quality of life had deteriorated because of pain, and 40% reported conflicts at school.

In the comprehensive care of these patients, the adolescent should be more involved in the decision-making and counseled on the time it will approximately take to achieve satisfactory results, as well as the obstacles and/or physical discomfort he or she may have to face. An interdisciplinary approach is recommended to help the patients cope with the possible psychosocial effects of the brace wearing.
